# Clinical validation of AWGC-defined cachexia in gastric cancer patients: impact on body composition and quality of life

**DOI:** 10.3389/fnut.2025.1659669

**Published:** 2025-10-27

**Authors:** Yan Huang, Xi Wu, Yan Ge, Chen Wang, Xin Ding, Junbo Zuo

**Affiliations:** ^1^Department of Clinical Nutrition, The Affiliated People’s Hospital of Jiangsu University, Zhenjiang, China; ^2^Department of Cardiovascular Medicine, The Affiliated People’s Hospital of Jiangsu University, Zhenjiang, China; ^3^Department of Geriatrics, The Affiliated People’s Hospital of Jiangsu University, Zhenjiang, China; ^4^Department of General Surgery, The Affiliated People’s Hospital of Jiangsu University, Zhenjiang, China

**Keywords:** cancer cachexia, the Asian Working Group for Cachexia, body composition, quality of life, gastric cancer

## Abstract

**Background:**

The Asian Working Group for Cachexia (AWGC) has released consensus criteria for diagnosing cachexia in Asians. Nevertheless, there is limited data regarding the application of these criteria in cancer patients. This study aimed to assess the changes in body composition and quality of life in gastric cancer (GC) patients with cachexia defined by the AWGC criteria.

**Methods:**

Body composition parameters were analyzed using CT images at the level of the third lumbar vertebra. The European Organization for Research and Treatment of Cancer Quality-of-Life Questionnaire-Core 30 (EORTC QLQ-C30) was used to evaluate health-related quality of life (HRQoL). The diagnosis of cachexia was according to the AWGC criteria and Fearon’s criteria.

**Results:**

A total of 431 patients with GC was included in this study. Among them, 160 patients (37.1%) were diagnosed with cachexia according to the AWGC criteria and 166 patients (38.5%) were diagnosed with cachexia based on the Fearon’s criteria. The agreement between the two criteria for diagnosing cachexia was moderate (*k* = 0.477, *p* < 0.001). Patients with AWGC-cachexia had significantly lower skeletal muscle index (SMI), visceral fat index (VFI), and subcutaneous fat index (SFI) compared to those without (*p* < 0.001). The prevalence of poor HRQoL was notably higher in patients with AWGC-defined cachexia (78.12% vs. 33.21%, *p* < 0.001). Furthermore, AWGC-defined cachexia was independently associated with poor HRQoL (OR = 5.92, 95% CI: 3.27–10.73; *p* < 0.001), while Fearon-defined cachexia did not show such an association (OR = 1.51, 95% CI: 0.86–2.65; *p* = 0.154).

**Conclusion:**

Patients with AWGC-defined cachexia showed significant changes in body composition and was independently associated with poor HRQoL.

## 1 Introduction

Gastric cancer (GC) is a common gastrointestinal malignancy. In 2022, there were more than 968,000 new GC cases and nearly 660,000 deaths, ranking it fifth in both cancer-related incidence and mortality worldwide (1). The highest incidence rates are found in Eastern Asia, and nearly half of all new cases and deaths each year occur in China (1, 2). In China, although significant progress has been made in GC screening, diagnosis, and treatment, more than 80% of patients are diagnosed at an advanced stage, and the overall 5-year survival rate is only 35.1% (3). Therefore, the prevention and treatment of GC present a great challenge to the public health system and lead to a severe social and economic burden.

Due to late diagnosis, tumor-induced inflammation, gastrointestinal dysfunction, and poor treatment outcomes, and other contributing factors, patients with GC are more prone to cancer-associated malnutrition with inflammation, also known as cancer cachexia (4). According to the international consensus established in 2011, cancer cachexia is defined as a persistent loss of skeletal muscle mass that cannot be completely reversed through conventional nutritional support, ultimately resulting in progressive functional impairment (5). Cachexia is observed in approximately 50-80% of cancer patients and is closely associated with a decline in quality of life, an increased risk of complications, and elevated cancer-related mortality rates (6, 7). Although the international diagnostic criteria for cachexia have been widely applied in clinical practice, they may not be suitable for Asian patients with different body types and ethnic backgrounds (8). In order to address this issue, the Asian Working Group for Cachexia (AWGC) has proposed a new diagnostic framework for diagnosing cancer cachexia in Asia (8). The diagnostic criteria for cachexia by the AWGC include weight loss, body mass index (BMI), and the presence of at least one of the following three symptoms: anorexia, decreased handgrip strength (HGS), or elevated levels of C-reactive protein (CRP) (8).

Recently, cachexia defined by the AWGC criteria has been shown to be closely associated with the prognosis of cancer patients. Sakaguchi et al. reported that the prevalence of cachexia defined by the AWGC criteria was 76%, which has significant prognostic value for Japanese patients with advanced cancer receiving palliative care (9). Xie et al. demonstrated that AWGC-defined cachexia serves as a straightforward and effective tool for predicting survival outcomes and medical burden in Chinese cancer patients (10). However, due to the limited number of studies in clinical settings, there is still a lack of sufficient data regarding its application, and its practical implementation has not yet been fully established.

Cancer patients with cachexia usually exhibit changes in body composition. It is worth noting that the AWGC criteria for the diagnosis of cachexia do not incorporate an assessment of body composition. As a result, the changes in body composition among patients with cachexia defined by the AWGC criteria need to be further investigated. Furthermore, no studies have explored the relationship between AWGC-defined cachexia and health-related quality of life (HRQoL) in cancer patients. Therefore, the aim of this study was to evaluate the changes in body composition and quality of life in GC patients with AWGC-defined cachexia.

## 2 Materials and methods

### 2.1 Study design and patients

This cross-sectional study involved a cohort of consecutive patients diagnosed with GC from October 2021 to July 2023 in our institution. All patients had been pathologically diagnosed with GC through gastroscopic biopsy prior to admission. The age of the participants ranged from 18 to 80 years, and none had a history of neoadjuvant therapy. Exclusion criteria were as follows: (1) lack of abdominal CT scans performed at our institution; (2) inability to complete questionnaires or assessments related to muscle strength; (3) a history of other malignancies within the past five years; (4) presence of severe comorbidities, such as active infections, heart failure, liver cirrhosis, or chronic kidney disease. Participants gave informed consent for the collection and analysis of their data. The study adhered to the Declaration of Helsinki guidelines and received approval from the Ethics Committee of The Affiliated People’s Hospital of Jiangsu University (Approval No. K-2025149-Y).

### 2.2 Data collection

The collected data included the following: age, sex, weight change (calculated as the difference between the the patient’s self-reported body weight at admission and their current body weight at 6 months prior to admission), BMI, Charlson Comorbidity Index (CCI) score, tumor-node-metastasis (TNM) stage, Eastern Cooperative Oncology Group performance status (ECOG PS), Nutritional Risk Screening-2002 (NRS-2002) score, HGS, CRP, Neutrophil-to-lymphocyte ratio (NLR: calculated as neutrophil count divided by lymphocyte count), hypoproteinemia (defined as a serum albumin level < 35 g/L), and anemia (defined as a hemoglobin level of < 120 g/L in males and < 110 g/L in females).

### 2.3 Body composition analysis

As described in our previous study, body composition was analyzed using CT images at the L3 level with Slice-O-Matic software version 5.0 (Tomovision, Magog, QC, Canada) (11). The tissue-specific Hounsfield unit (HU) thresholds for differentiating muscle from fat are as follows: −29 to + 150 HU for skeletal muscle, −150 to −50 HU for visceral fat, and −190 to −30 HU for subcutaneous fat. Subsequently, the cross-sectional areas at the L3 level (cm^2^) were normalized by height squared (m^2^) to calculate the skeletal muscle index (SMI, cm^2^/m^2^), visceral fat index (VFI, cm^2^/m^2^), and subcutaneous fat index (SFI, cm^2^/m^2^). The VFI-to-SFI ratio (VSR) was obtained by calculating the quotient of VFI and SFI. The mean tissue attenuation values (HU) of the entire cross-sectional area at the L3 level on CT images were used to assess skeletal muscle radiodensity (SMD), visceral adipose radiodensity (VAD), and subcutaneous adipose radiodensity (SAD). Low muscle mass was defined as L3-SMI ≤ 40.8 cm^2^/m^2^ in males and ≤ 34.9 cm^2^/m^2^ in females (12).

### 2.4 HRQoL assessment

Health-related quality of life was evaluated by means of the European Organization for Research and Treatment of Cancer (EORTC) Quality-of-Life Questionnaire-Core 30 (EORTC QLQ-C30) (13). The summary score was obtained by averaging the 13 subscales of the QLQ-C30, excluding the financial impact and global quality of life (14). The calculation formula for this score is: [(physical functioning) + (role functioning) + (emotional functioning) + (cognitive functioning) + (social functioning) + (100−fatigue) + (100−nausea and vomiting) + (100−pain) + (100−dyspnea) + (100−insomnia) + (100−loss of appetite) + (100−constipation) + (100−diarrhea)]/13. Those patients whose scores were lower than the median of the summary score were categorized as having poor HRQoL (15).

### 2.5 Diagnosis of cachexia

As shown in Table 1, the criteria for Fearon-defined cachexia are as follows: weight loss > 5% over the past 6 months (in the absence of simple starvation), or BMI < 20 kg/m^2^ with weight loss > 2%, or low muscle mass with weight loss > 2% (5). The criteria for AWGC-defined cachexia are: weight loss > 2% within 3–6 months or BMI < 21 kg/m^2^, along with one or more of the following: (1) anorexia; (2) reduced HGS; (3) elevated CRP (8). Anorexia was assessed by means of the EORTC QLQ-C30 anorexia symptom scale. This scale encompasses response options such as “Not at all,” “A little,” “Quite a bit,” and “Very much.” A response of “Not at all” indicates the non-existence of anorexia symptoms (16). In this study, a patient was considered to have anorexia if they replied with “A little,” “Quite a bit,” or “Very much.”

**TABLE 1:** Classification of diagnostic criteria for cachexia.

Syndrome	Diagnosis	Cut offs
Fearon-defined cachexia	1. Weight loss > 5% over the past 6 months (in absence of simple starvation); or 2. BMI < 20 kg/m^2^ with weight loss > 2%; or 3. Low muscle mass with weight loss > 2%	Low muscle mass: L3 SMI ≤ 40.8 cm^2^/m^2^ in males and ≤ 34.9 cm^2^/m^2^ in females
AWGC-defined cachexia	weight loss > 2% within 3–6 months or BMI < 21 kg/m^2^, along with one or more of the following: (1) anorexia; (2) reduced HGS; (3) elevated CRP	Reduced HGS: < 28.0 kg for male and < 18.0 kg for female Elevated CRP: > 5 mg/L

AWGC, Asian Working Group for Cachexia; BMI, body mass index; CRP, C-reactive protein; HGS, handgrip strength.

### 2.6 Statistical analysis

Statistical analyses were carried out using SPSS version 25.0 (IBM Corp, Armonk, NY, United States). Statistical significance was defined as *p* < 0.05. Categorical variables were expressed as numbers accompanied by percentages and analyzed through the chi-squared test. Continuous data were reported as either means with standard deviation (SD) or medians with inter-quartile range (IQR), determined through statistical assessments of normality (assessed using the Kolmogorov–Smirnov test). Subsequently, independent t-tests or Mann-Whitney *U*-tests were, respectively conducted for further analyses. To identify potential risk factors associated with poor HRQoL, univariate logistic regression analyses were first carried out. Subsequently, variables that demonstrated a *p*-value of less than 0.1 in the univariate analysis and exhibited no multicollinearity (with a Variance Inflation Factor below 10) were selected for the multivariate analysis. The Kappa coefficient (k) was employed to evaluate the agreement between cachexia defined by the AWGC criteria and the Fearon’s criteria.

## 3 Results

### 3.1 Baseline patient characteristics

**FIGURE 1:**
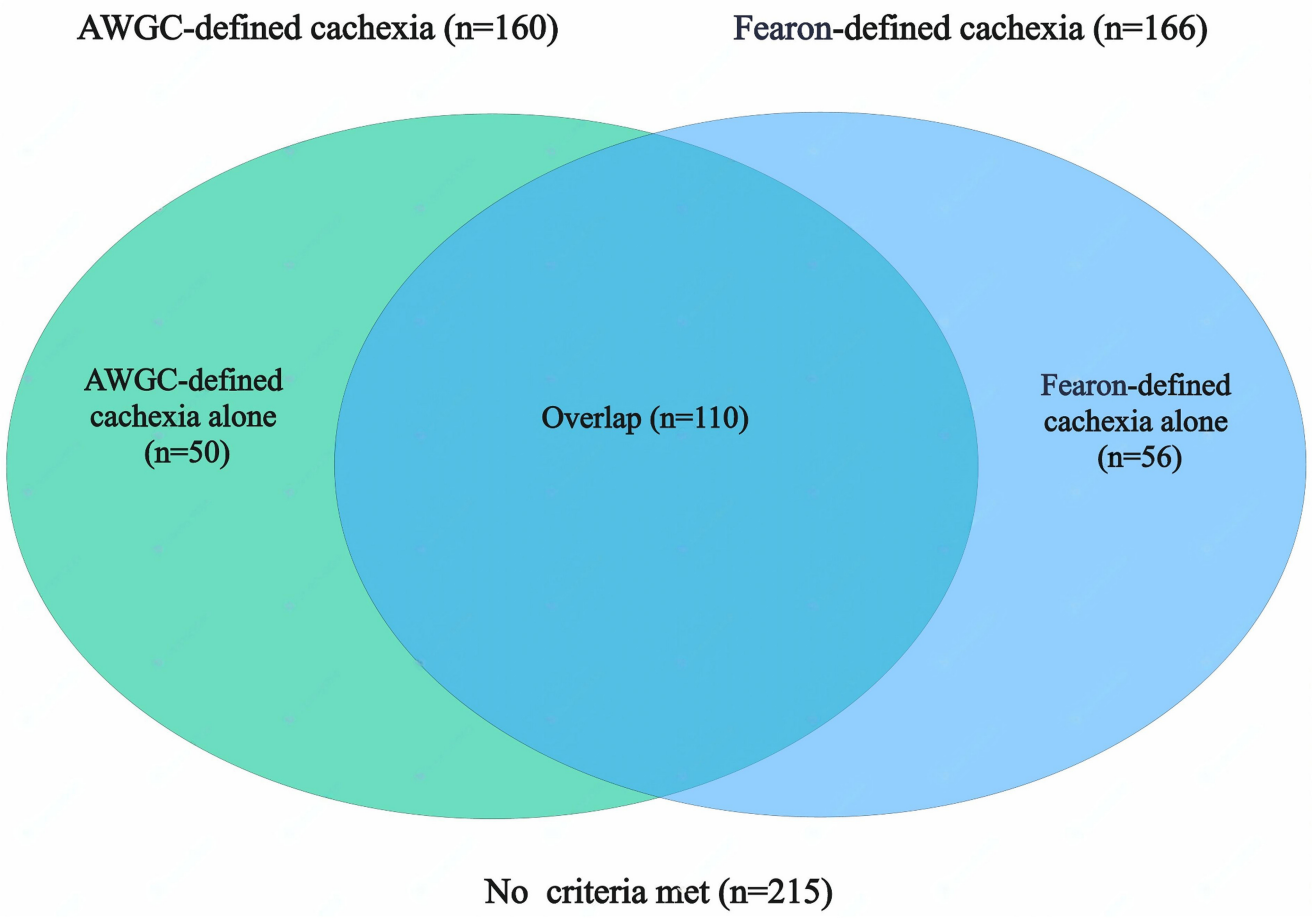
Co-occurrence of cachexia identified by Fearon’s and AWGC criteria in patients with gastric cancer (GC). AWGC, Asian Working Group for Cachexia.

From October 2021 to July 2023, a total of 474 patients diagnosed with GC were included in the study. Among these patients, nine patients were excluded due to the lack of abdominal CT scans performed at our hospital, eight patients were unable to complete questionnaires or muscle strength assessments, 12 had a history of other malignancies within the last 5 years, and 14 presented with severe comorbidities. After excluding these cases, a final cohort of 431 patients was available for analysis (Supplementary Figure 1). The baseline patient characteristics are shown in Table 2. Among the patients, 309 (71.69%) were male and 122 (28.31%) were female. The median age was 68 years, and the mean BMI was 23.44 ± 3.33 kg/m^2^. Based on the NRS-2002 nutritional screening tool, 279 patients (64.73%) were found to be at risk of malnutrition (NRS-2002 score ≥ 3). Using the AWGC criteria for further diagnosis, 257 patients (59.63%) had involuntary weight loss (> 2%), 101 patients (23.43%) had a low BMI (< 21 kg/m^2^), 78 patients (18.10%) showed low HGS, and 160 patients (37.12%) had anorexia. In total, 160 patients (37.12%) were diagnosed with cachexia according to the AWGC criteria. Additionally, based on the Fearon’s criteria, 166 patients (38.52%) were diagnosed with cachexia. Among these patients, 110 patients (25.52%) met both the AWGC-cachexia criteria and the Fearon-cachexia criteria, 50 patients (11.60%) met only the AWGC-cachexia criteria, 56 patients (12.99%) met only the Fearon-cachexia criteria, and 215 patients (49.88%) did not meet any cachexia criteria (Figure 1). The agreement between the two criteria for diagnosing cachexia was moderate (*k* = 0.477, *p* < 0.001).

**TABLE 2:** Baseline characteristics of study patients based on AWGC-defined cachexia.

Variables	Total (*n* = 431)	With AWGC-defined cachexia (*n* = 160)	Without AWGC-defined cachexia (*n* = 271)	*P*-value
Sex, *n* (%)	309 (71.69)	117 (73.12)	192 (70.85)	0.612
Male				–
Female	122 (28.31)	43 (26.88)	79 (29.15)	–
Age, years	68 (62, 72)	69 (61.75, 72.25)	68 (62, 72)	0.487
BMI, kg/m^2^	23.44 ± 3.33	22.13 ± 3.08	24.21 ± 3.23	< 0.001
BMI < 21 kg/m^2^	101 (23.43)	57 (35.62)	44 (16.24)	< 0.001
Weight loss, %	3.20 (0.00, 6.38)	6.24 (4.18, 9.46)	0.00 (0.00, 3.87)	< 0.001
Weight loss ≥ 2%, *n* (%)	257 (59.63)	149 (93.12)	108 (39.85)	< 0.001
NRS-2002 score ≥ 3, *n* (%)	279 (64.73)	143 (89.38)	136 (50.18)	< 0.001
Anorexia, *n* (%)	160 (37.12)	125 (78.12)	35 (12.92)	< 0.001
CCI score, *n* (%)				0.748
0	327 (75.87)	120 (75.00)	207 (76.38)	–
1	74 (17.17)	30 (18.75)	44 (16.24)	–
≥ 2	30 (6.96)	10 (6.25)	20 (7.38)	–
ECOG PS score, *n* (%)				< 0.001
0	254 (58.93)	65 (40.62)	189 (69.74)	–
1	126 (29.23)	60 (37.50)	66 (24.35)	–
≥ 2	51 (11.83)	35 (21.88)	16 (5.90)	–
TNMstage, *n* (%)				< 0.001
I	104 (24.13)	15 (9.38)	89 (32.84)	–
II	82 (19.03)	36 (22.50)	46 (16.97)	–
III	186 (43.16)	75 (46.88)	111 (40.96)	–
IV	59 (13.69)	34 (21.25)	25 (9.23)	–
CRP > 5 mg/L, *n* (%)	95 (22.04)	65 (40.62)	30 (11.07)	< 0.001
NLR	2.47 (1.89, 3.49)	3.08 (2.15, 4.30)	2.29 (1.74, 3.09)	< 0.001
Anemia, *n* (%)	183 (42.46)	97 (60.62)	86 (31.73)	< 0.001
Hypoproteinemia, *n* (%)	128 (29.70)	72 (45.00)	56 (20.66)	< 0.001
HGS, kg	31.52 ± 8.50	28.85 ± 7.99	33.09 ± 8.42	< 0.001
Low HGS, *n* (%)	78 (18.10)	62 (38.75)	16 (5.90)	< 0.001
Fearon-defined cachexia, *n* (%)	166 (38.51)	110 (68.75)	56 (20.66)	< 0.001

AWGC, Asian Working Group for Cachexia; BMI, body mass index; CCI, Charlson Comorbidity Index; CRP, C-reactive protein; ECOG PS, Eastern Cooperative Oncology Group performance status; HGS, handgrip strength; NLR, neutrophil-to-lymphocyte ratio; NRS-2002, Nutritional Risk Screening-2002; TNM, tumor–node–metastasis.

### 3.2 Comparison of patients with or without AWGC-defined cachexia

As shown in Table 2, patients with AWGC-defined cachexia had lower BMI (22.13 ± 3.08 vs. 24.21 ± 3.23; *p* < 0.001), higher weight loss proportion [6.24 (IQR 4.18–9.46) vs. 0.00 (0.00–3.87); *p* < 0.001], and higher NLR [3.08 (2.15–4.30) vs. 2.29 (1.74–3.09); *p* < 0.001]. No difference was observed in sex distribution (*p* = 0.612) or CCI score (*p* = 0.748). Patients with AWGC-defined cachexia had higher proportions of hypoalbuminemia (45.00% vs. 20.66%; *p* < 0.001), CRP > 5 mg/L (40.62% vs. 11.07%; *p* < 0.001), anemia (60.62% vs. 31.73%; *p* < 0.001), low grip strength (38.75% vs. 5.90%; *p* < 0.001), and anorexia (78.12% vs. 12.92%; *p* < 0.001). Differences were also observed in ECOG PS (*p* < 0.001) and TNM stage (*p* < 0.001) between patients with and without AWGC-defined cachexia.

### 3.3 Changes in body composition in patients with AWGC-defined cachexia

The results regarding the changes in the body composition evaluated through CT were presented in Table 3. Patients with AWGC-cachexia had significantly lower SMI (42.44 ± 7.20 vs. 46.46 ± 7.38; *p* < 0.001), lower SFI [31.87 (20.87–46.23) vs. 41.59 (29.78–56.44); *p* < 0.001], and lower VFI [32.44 (18.44–52.48) vs. 47.16 (29.96–70.23); *p* < 0.001]. SMD was also lower in patients with cachexia [35.89 (31.40–40.02) vs. 37.52 (32.80–41.25); *p* = 0.031]. Furthermore, patients with cachexia exhibited higher SAD [−95.40 (−107.09 to −69.86) vs. −105.40 (−128.18 to −94.11); *p* < 0.001] and higher VAD [−90.20 (−108.47 to −66.69) vs. −100.40 (−138.20 to −89.79); *p* < 0.001]. The prevalence of low muscle mass was also higher in patients with cachexia (31.25% vs. 14.02%; *p* < 0.001). No statistically significant difference was observed in VSR between two groups (*p* = 0.213).

**TABLE 3:** Changes in body composition in patients with AWGC-defined cachexia.

Variables	Total (*n* = 431)	With cachexia (*n* = 160)	Without cachexia (*n* = 271)	*P*-value
SMI, cm^2^/m^2^	44.97 ± 7.56	42.44 ± 7.20	46.46 ± 7.38	< 0.001
SFI, cm^2^/m^2^	38.04 (25.82, 53.06)	31.87 (20.87, 46.23)	41.59 (29.78, 56.44)	< 0.001
VFI, cm^2^/m^2^	41.46 (24.26, 63.12)	32.44 (18.44, 52.48)	47.16 (29.96, 70.23)	< 0.001
VSR	1.01 (0.72, 1.50)	0.95 (0.69, 1.38)	1.06 (0.72, 1.52)	0.213
SMD, HU	36.97 (32.04, 40.68)	35.89 (31.40, 40.02)	37.52 (32.80, 41.25)	0.031
SAD, HU	−102.30 (−117.30, −82.78)	−95.40 (−107.09, −69.86)	−105.40 (−128.18, −94.11)	< 0.001
VAD, HU	−97.43 (−125.50, −82.58)	−90.20 (−108.47, −66.69)	−100.40 (−138.20, −89.79)	< 0.001
Low muscle mass, *n* (%)	88 (20.42)	50 (31.25)	38 (14.02)	< 0.001

AWGC, Asian Working Group for Cachexia; SAD, subcutaneous adipose radiodensity; SMD, skeletal muscle radiodensity; SMI, skeletal muscle index; SFI, subcutaneous fat index; VAD, visceral adipose radiodensity; VFI, visceral fat index; VSR, the VFI-to-SFI ratio.

### 3.4 Relationship between cachexia and HRQoL

**FIGURE 2:**
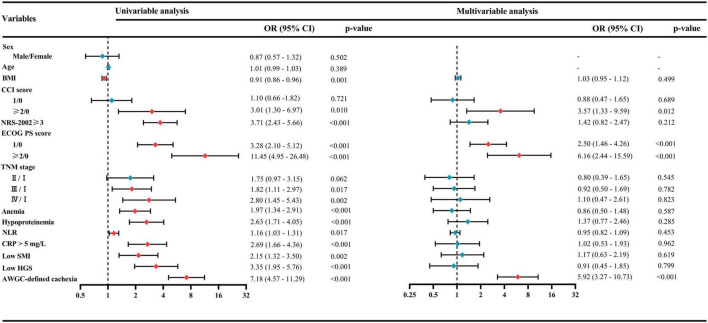
Univariate and multivariate logistic regression analyses for identifying risk factors associated with poor health-related quality of life (HRQoL). AWGC, Asian Working Group for Cachexia; BMI, body mass index; CCI, Charlson Comorbidity Index; CRP, C-reactive protein; ECOG PS, Eastern Cooperative Oncology Group performance status; HGS, handgrip strength; NLR, neutrophil-to-lymphocyte ratio; NRS-2002, Nutritional Risk Screening-2002; SMI, skeletal muscle index; TNM, tumor–node–metastasis.

As presented in Table 4, the summary score was significantly lower in patients with AWGC-defined cachexia [79.5 (66.3–88.3)] compared to those without [92.3 (86.2–96.2); *p* < 0.001]. The prevalence of poor HRQoL was notably higher in patients with AWGC-defined cachexia (78.12% vs. 33.21%, *p* < 0.001). Furthermore, patients with AWGC-defined cachexia exhibited poorer global health status, along with reduced physical function, role function, emotional function, cognitive function, and social function (all *p* < 0.05). In addition, patients with AWGC-defined cachexia reported more severe symptoms including fatigue, nausea and vomiting, pain, dyspnea, insomnia, loss of appetite, and diarrhea (all *p* < 0.05), except for constipation (*p* = 0.061). Univariate and multivariate logistic regression analyses revealed that AWGC-defined cachexia had a 5.92-fold increased risk (95%CI: 3.27–10.73; *p* < 0.001) of developing poor HRQoL compared to those without cachexia (Figure 2). In contrast, Fearon-defined cachexia was not identified as an independent risk factor for poor HRQoL (OR = 1.51, 95% CI: 0.86–2.65; *p* = 0.154, Supplementary Figure 2).

**TABLE 4:** Comparison of HRQoL in patients with or without AWGC-defined cachexia.

Variables	With AWGC-defined cachexia (*n* = 160)	Without AWGC-defined cachexia (*n* = 271)	*P*-value
Summary score	79.52 (66.27, 88.28)	92.31 (86.18, 96.15)	< 0.001
Poor HRQoL, *n* (%)	125 (78.12)	90 (33.21)	< 0.001
Global health status score	50.00 (33.33, 66.67)	83.33 (58.33, 100.00)	< 0.001
**Function score**			
Physical functioning	86.67 (66.67, 93.33)	100.00 (86.67, 100.00)	< 0.001
Role functioning	83.33 (66.67, 100.00)	100.00 (83.33, 100.00)	< 0.001
Emotional functioning	83.33 (66.67, 100.00)	91.67 (75.00, 100.00)	< 0.001
Cognitive functioning	83.33 (66.67, 100.00)	100.00 (83.33, 100.00)	< 0.001
Social functioning	83.33 (66.67, 100.00)	100.00 (66.67, 100.00)	< 0.001
**Symptoms score**			
Fatigue	33.33 (11.11, 47.22)	0.00 (0.00, 22.22)	< 0.001
Nausea and vomiting	16.67 (0.00, 33.33)	0.00 (0.00, 0.00)	< 0.001
Pain	16.67 (0.00, 50.00)	0.00 (0.00, 16.67)	< 0.001
Dyspnea	0.00 (0.00, 33.33)	0.00 (0.00, 33.33)	< 0.001
Insomnia	33.33 (0.00, 33.33)	0.00 (0.00, 33.33)	< 0.001
Loss of appetite	33.33 (33.33, 66.67)	0.00 (0.00, 0.00)	< 0.001
Constipation	0.00 (0.00, 33.33)	0.00 (0.00, 0.00)	0.061
Diarrhea	0.00 (0.00, 33.33)	0.00 (0.00, 0.00)	0.046

AWGC, Asian Working Group for Cachexia; HRQoL, health-related quality of life.

## 4 Discussion

To the best of our knowledge, this study is one of the few to comprehensively investigate the body composition and quality of life of cancer cachexia patients based on the AWGC criteria. Our findings showed that patients with AWGC-defined cachexia exhibited significantly lower muscle mass and fat mass compared to those without cachexia. Furthermore, the prevalence of poor HRQoL was markedly higher among patients with AWGC-defined cachexia. Additionally, AWGC-defined cachexia was found to be independently associated with poor HRQoL. These results highlight the reliability of the AWGC diagnostic criteria for the Asian population and emphasize the critical importance of early identification and intervention of cachexia to enhance the quality of life for cancer patients.

It is known that the current international criteria for the diagnosis of cachexia are based on thresholds originating from Western populations, which may not be applicable to Asians owing to differences in body composition (8, 17). In response to this concern, the AWGC was established with the aim of reaching an agreement on diagnostic criteria and important clinical outcomes specific to cachexia in Asia (8). There are significant differences in the diagnosis of cachexia between the AWGC criteria and the Fearon’s criteria. Firstly, the AWGC criteria are more lenient and flexible, enabling the diagnosis of cachexia through the unrestricted combination of multiple criteria. Secondly, the AWGC criteria do not directly assess muscle mass. Instead, they use grip strength as a proxy measure, which is simpler, more convenient, and easier to implement. Finally, the AWGC criteria include anorexia and inflammatory indicators, which are closely related to the formation of cachexia. However, it should be noted that the AWGC criteria rely on patient-reported weight loss and anorexia, which are susceptible to recall bias and may compromise diagnostic accuracy. Furthermore, although HGS is a validated measure of overall strength and is correlated with muscle mass, it may not precisely capture the specific quantitative and qualitative changes in skeletal muscle compared to CT-derived metrics like the SMI or SMD. Zhang et al. conducted a comparative analysis of the AWGC criteria and Fearon’s criteria to assess differences in clinical outcomes among patients with curable GC. Their findings indicated that the AWGC criteria demonstrated stronger correlations with postoperative complications and overall survival compared to Fearon’s criteria in GC patients (18). Furthermore, our results showed that patients with AWGC-defined cachexia exhibited a significantly higher risk of poor HRQoL compared to those without cachexia. In contrast, Fearon-defined cachexia did not show such an association. Therefore, these findings validate the reliability of the AWGC criteria for the diagnosis of cachexia in Asian populations.

Cachexia is prevalent among patients with GC. This syndrome is characterized by the loss of body composition, specifically skeletal muscle and adipose tissue (5). It is mainly caused by diminished food intake and metabolic alterations, such as increased energy expenditure, heightened catabolism, and inflammatory responses (7). In this study, we conducted a comparative analysis of body composition differences between patients with cachexia and those without. Our findings revealed a significant reduction in both muscle mass and fat mass among patients suffering from cachexia. Furthermore, there was a notable decrease in skeletal muscle density, while fat density exhibited a significant increase. It is essential to highlight that reduced radiodensity values in skeletal muscle or increased radiodensity values in adipose tissue are regarded as pathological. Lower skeletal muscle radiodensity can reflect intramuscular fat infiltration, reduced physical function, and metabolic dysregulation (19). On the other hand, higher fat tissue radiodensity may suggest increased lipid consumption and a stronger inflammatory response, which has been shown to be associated with increased mortality in cancer patients (20, 21). Thus, although the assessment of body composition is not explicitly included in the AWGC criteria for diagnosing cachexia, significant loss of body composition can be observed in patients with AWGC-defined cachexia.

Malnutrition and cachexia are both recognized as nutritional disorders characterized by a common feature of reduced fat-free mass. Despite showing conceptual overlap, they have distinct characteristics, etiologies, and therapeutic approaches (22). Currently, the Global Leadership Initiative on Malnutrition (GLIM) criteria are widely used in clinical practice for the diagnosis of malnutrition (23). The recently established AWGC-cachexia criteria show significant similarities to the GLIM-malnutrition criteria. Both sets of criteria include common elements like weight loss, low BMI, decreased appetite, muscle assessment, and elevated inflammation levels. Wang et al. reported that cachexia and malnutrition both served as independent risk factors for postoperative complications and overall survival in patients with GC. Patients with both conditions tended to have a worse prognosis. Their results indicated that AWGC-defined cachexia and GLIM-defined malnutrition served as two distinct and complementary tools for evaluating prognosis in preoperative nutritional assessment (24). In our study, a significant proportion of patients with AWGC-defined cachexia also met the criteria for GLIM-defined malnutrition (Supplementary Figure 3). Malnutrition diagnosed according to the GLIM criteria has been shown to be independently associated with poorer quality of life in cancer patients (15). The strong association we found between AWGC-defined cachexia and poor HRQoL may thus be partly explained by this overlap, as the cachexia criteria encompass key elements of nutritional and inflammatory deterioration that directly impact a patient’s wellbeing.

Our results showed that patients with AWGC-defined cachexia had significantly lower summary scores (79.5 vs. 92.3) and global health status (50.0 vs. 83.3) on the EORTC QLQ-C30 compared to non-cachectic patients. Additionally, the mean change in scores in most functional scales and symptom scales was greater than 10. Although these differences are statistically significant, their clinical relevance is further highlighted when interpreted using established Minimal Clinically Important Differences (MCIDs). For the EORTC QLQ-C30, a change of greater than 10 can be regarded as moderate or large changes in quality of life (25). Additionally, in a multivariable logistic regression analysis adjusted for confounding factors, we observed that patients with AWGC-defined cachexia exhibited a significantly high risk of poor HRQoL. We proposed several reasonable explanations for the effectiveness of the AWGC criteria as a predictive tool for quality of life. Firstly, anorexia is one of the diagnostic criteria for cancer cachexia and an indicator for assessing quality of life. It can lead to a reduction in food intake, typically caused by digestive system symptoms such as nausea, vomiting, and diarrhea. These symptoms are crucial for the assessment of quality of life. Secondly, weight loss and low BMI, used as diagnostic criteria for cachexia, have also been confirmed to be closely related to poor quality of life (26, 27). Moreover, Our CT-based body composition analysis showed that patients with AWGC-defined cachexia had a significantly lower skeletal muscle mass compared to non-cachectic patients. This reduction in muscle mass directly translates to impaired physical capacity. Thirdly, low grip strength, as one of the criteria for diagnosing cachexia, can indicate impaired muscle function and reflect a decline in physical function. Finally, the elevation of CRP, one of the criteria for diagnosing cachexia, reflects the systemic inflammatory condition and has been reported in many diseases to be associated with impaired quality of life (28, 29). Thus, the AWGC criteria could serve as a useful tool for assessing HRQoL.

It is essential to take into account the limitations of the current study. Firstly, this study adopted a cross-sectional design, which means that the causal direction of the observed association cannot be determined. Secondly, this study was conducted at a single center and only involved Chinese patients with GC. Therefore, further investigations are needed to verify whether the cachexia defined by AWGC criteria can be applied to other disease types and populations in different Asian countries. Thirdly, the assessment of cachexia was carried out at a single time point after admission, lacking subsequent follow-up information. This may result in certain unreliability in evaluating the long-term impact of AWGC-defined cachexia.

In conclusion, the findings of the present study showed that nearly one-third of patients with GC at admission were found to have cachexia defined by the AWGC criteria. Patients with AWGC-defined cachexia exhibited significant alterations in body composition, including reductions in skeletal muscle mass and fat mass. Additionally, AWGC-defined cachexia was significantly associated with poorer quality of life in patients with GC. Our research further validated the reliability of the AWGC criteria for diagnosing cachexia in the Asian population.

## Data Availability

The raw data supporting the conclusions of this article will be made available by the authors, without undue reservation.
