# Supplementary material for: Clinical validation of AWGC-defined cachexia in gastric cancer patients: impact on body composition and quality of life
Source: Front Nutr. 2025 Oct 27;12:1659669. doi: 10.3389/fnut.2025.1659669 (PMC12597802; doi:10.3389/fnut.2025.1659669)
Supplement: Supplementary file 1 [file Data_Sheet_1.pdf]

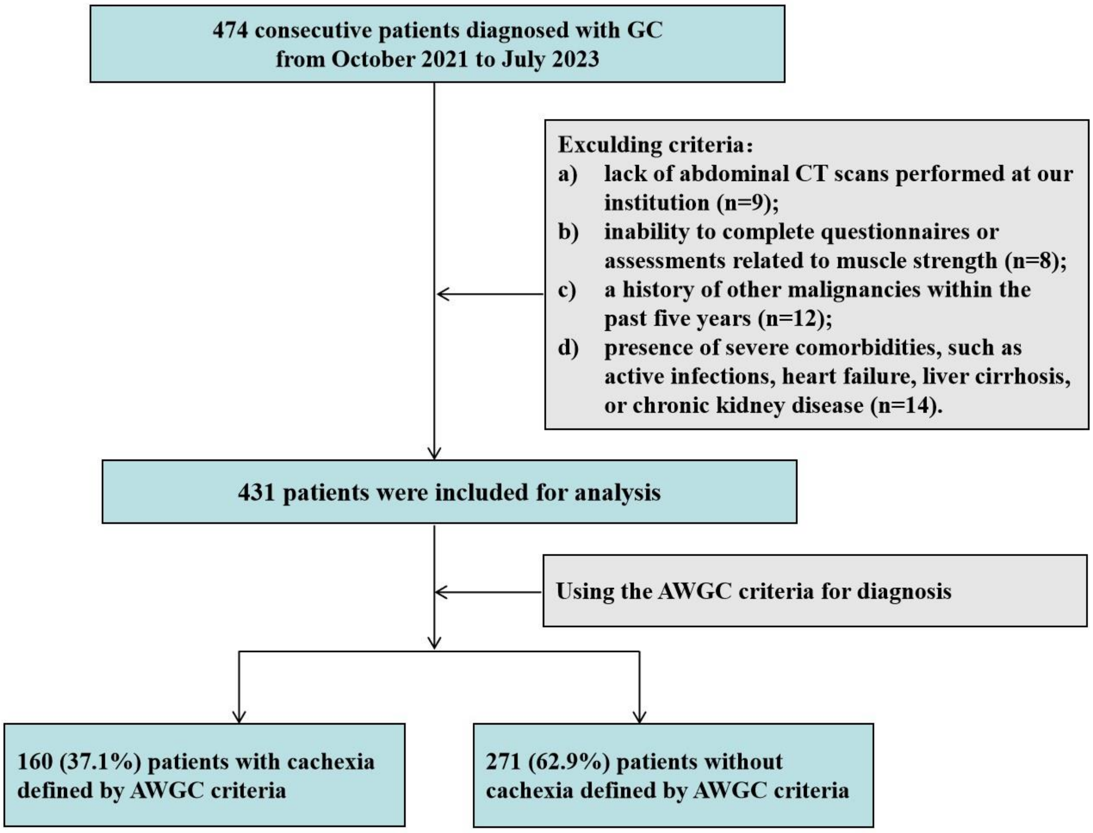

Figure S1. Flow chart of patient selection. AWGC, Asian Working Group for Cachexia; CT, computed tomography; GC, gastric cancer.

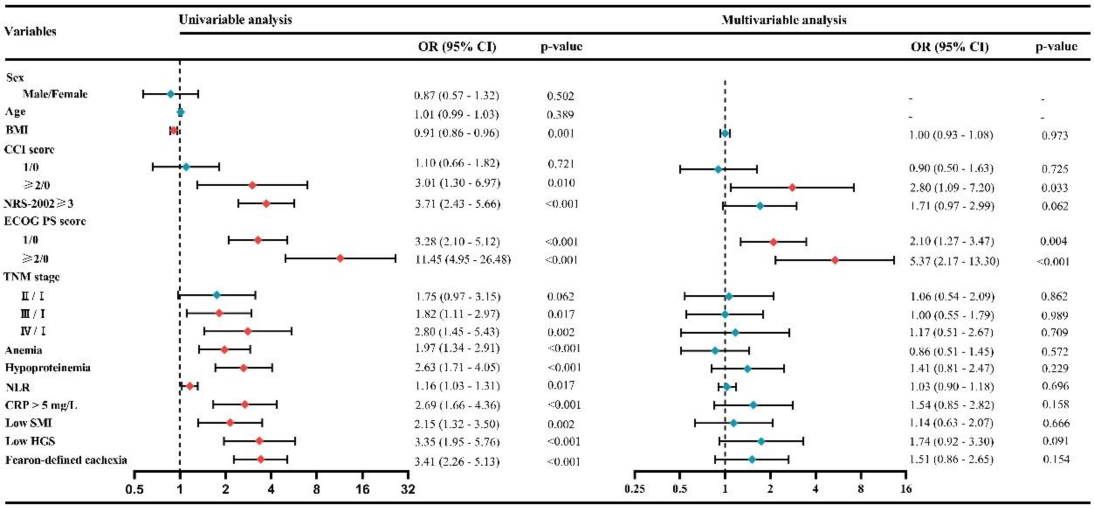

Figure S2. Univariate and multivariate logistic regression analyses for identifying risk factors associated with poor health-related quality of life (HRQoL). BMI, body mass index; CCI, Charlson Comorbidity Index; CRP, C-reactive protein; ECOG PS, Eastern cooperative oncology group performance status; HGS, Handgrip strength; NLR, neutrophil-to-lymphocyte ratio; NRS-2002, Nutritional Risk Screening-2002; SMI, skeletal muscle index; TNM, tumor–node–metastasis.

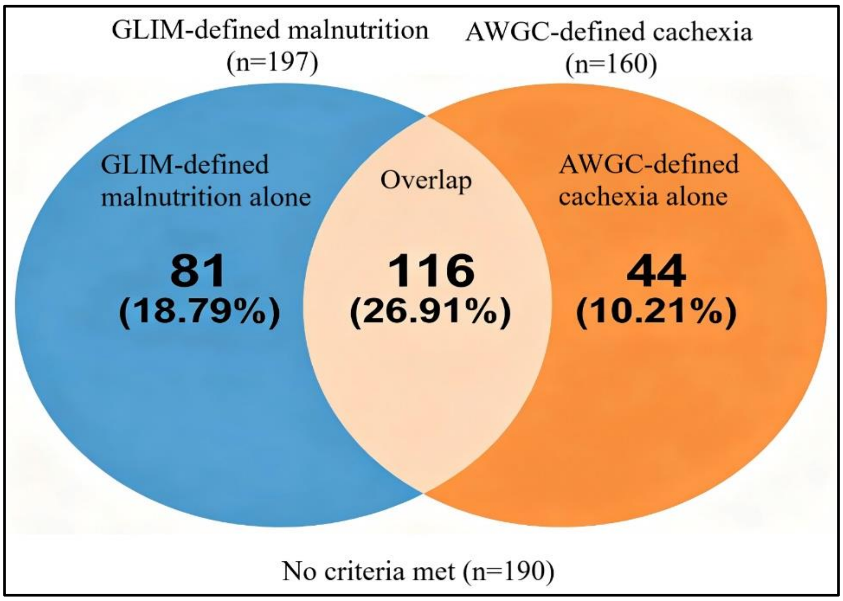

Figure S3. Co-occurrence of GLIM-defined malnutrition and AWGC-defined cachexia in patients with gastric cancer (GC). AWGC, Asian Working Group for Cachexia; GLIM, Global Leadership Initiative on Malnutrition.
